# Diagnosis and Treatment of Snake Envenomation in Dogs in Queensland, Australia

**DOI:** 10.3390/vetsci8020014

**Published:** 2021-01-20

**Authors:** Ludovica Valenza, Rachel Allavena, Mark Haworth, Jonathon Cochrane, Joerg Henning

**Affiliations:** School of Veterinary Science, University of Queensland, Gatton, QLD 4343, Australia; ludovalenza@hotmail.com (L.V.); r.allavena@uq.edu.au (R.A.); m.haworth@uq.edu.au (M.H.); jonno_vet@hotmail.com (J.C.)

**Keywords:** snake, envenomation, dogs, Australia

## Abstract

Australia has some of the most venous snakes in the world, and envenomations of domestic dogs are common, but clinical signs as well as the diagnostic procedures and treatments of snake envenomations are poorly described. Therefore, we invited veterinary clinics in the state of Queensland, Australia, to provide detailed data on snake envenomation cases in dogs. A total of 230 cases were reported from 19 veterinary hospitals, with an average of 12.1 dogs per clinic, per year. Detailed case data were provided from 20 dogs—of these, 65.0% (13/20) were envenomated during the daytime, with collapse and paresis being the most common signs reported by owners. The median time between the onset of clinical signs and admission to the veterinary hospital was 60 min. Clinical signs were the sole diagnostic modality utilised by veterinarians in 30.0% (6/20) of cases. Activated clotting time was the most common diagnostic procedure conducted, while snake venom detection kits (SVDK) were only used in 15.0% (3/20) of cases. Of the dogs that received antivenom (85.0%, 17/20), the tiger/multibrown combination (3000 units tiger/4000 units brown) was predominately (13/17) provided. Three of the 17 dogs that received antivenom (17.6%) died or were euthanised. About 82.4% (14/17) of the dogs treated with antivenom, but only 33.3% (1/3) of the dogs not treated with antivenom, recovered (*p* = 0.140). Overall, veterinarians relied frequently on medical history, clinical signs, and diagnostic tests other than the SVDK and, thus, most likely, administered snake envenomation treatment based on their clinical experience.

## 1. Introduction

Australia is home to some of the world’s deadliest terrestrial snakes. Of the estimated 140 snake species recognised, 92 possess venom glands [1]. With the exception of the brown tree snake, the venom-producing snakes belong to the family Elapidae, which deliver their venom through two hollow, fixed, front fangs. The Australian elapids of clinical importance are grouped into five major categories: brown snakes (*Pseudonaja*), tiger snakes (*Notechis*), black snakes (*Pseudechis*), death adders (*Acanthophis*), and taipans (*Oxyuranus*) [2].

It had been estimated that between 500 and 1000 humans are bitten by snakes in Australia every year [3,4]. Of 718 confirmed human snake bite cases reported between 2005 and 2015, 41% were caused by eastern brown snakes, 17% by tiger snakes, and 16% by red-bellied black snakes [5]. Reliable companion animal statistics are harder to acquire due to a lack of confirmed diagnosis. Research conducted more than 20 years ago estimated 6200 cases of snake envenomation in domestic species in Australia per year with 61% and 35% of these cases occurring in dogs and cats, respectively [6]. Although reports on snake envenomation in small animals have been published [7,8,9], the clinical signs reported by owners and treating veterinarians as well as the diagnostic procedures and treatments of elapid snake envenomation conducted by veterinarians in small animal practices are poorly described. Therefore, the objective of this research was to describe the frequency, clinical characteristics, diagnosis, treatments, and outcomes of dogs admitted with assumed elapid snake envenomation to veterinary clinics in Queensland, Australia.

## 2. Materials and Methods

Small and mixed animal veterinary clinics in Queensland, Australia, were targeted to collect data on snake envenomations in dogs. The platform “Survey Monkey” [10] was used to develop an electronic data collection form (a copy of the form is provided as Supplementary Figure S1). The form had two sections. Firstly, the frequency of snake envenomations over the period of one year was explored (January until December 2016), while in the second part, information on individual patients was collected, including the environment where the envenomation occurred, the clinical signs as observed by owners and recorded by veterinarians (including the time between onset of clinical signs and admission). Diagnostic tests and treatments performed by the veterinarian, the number of days of hospitalisation, and the outcome of hospitalisation were also recorded.

As no register of veterinary clinics was publicly available, a sampling frame was created by identifying 145 postal addresses of veterinary clinics in Queensland through Google searches. A postcard describing the project and providing the address to the project website [11], which contained a link to the data collection form, was sent out to these veterinary clinics. In addition, the Australian Veterinary Association [12] published the link to the data collection form in their monthly e-newsletter and on their Facebook site. Finally, a link to the data collection was posted on a professional Facebook group webpage for veterinarians and veterinary nurses in Queensland. Data collection was conducted between 1 April and 30 August 2017.

Data were summarised using descriptive statistics and cross-tabulations. The outcome of hospitalisation was compared between usage and non-usage of antivenom using the Fisher’s exact test. Results were considered significantly different at a *p*-value < 0.05.

## 3. Results

**Figure 1:**
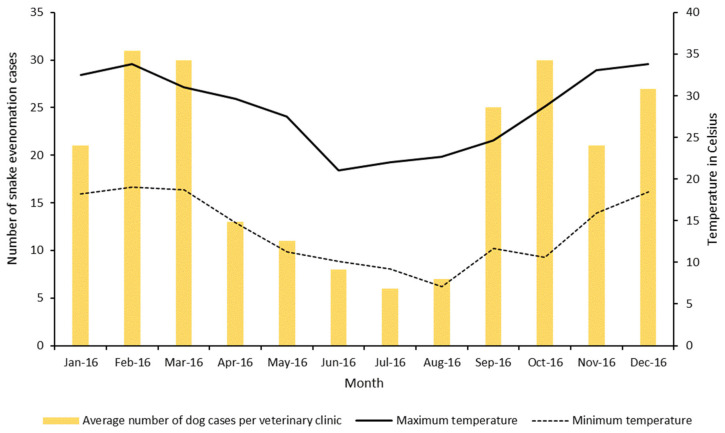
Average number of dogs diagnosed with snake envenomations per month across 19 veterinary clinics, in Queensland, Australia, in 2016 and mean minimum and maximum temperature per month in Queensland, Australia, in 2016 (temperature data were recorded by the Bureau of Meteorology station location 40082, University of Queensland, Gatton, Australia; latitude: −27.54, longitude: 152.34).

A total of 19 veterinary clinics completed the data collection form, reporting 230 envenomations in dogs over the 12-month period, with an average (range) of 12.1 (1–51) and a median of 11 dog envenomations reported per clinic, per year in Queensland, Australia, in 2016. The locations of the veterinary clinics that responded providing data are shown in Supplementary Figure S2. The peak months for snake envenomation were between September and March, corresponding with the warmer weather in Australia (Figure 1).

A complete history was provided for 20 dogs with snake envenomation, and 12 were females and eight were males. The majority of dog breeds affected were terriers (40.0%, 8/20, including four Jack Russell, two Fox, two Staffordshire Bull, one Boston, and one West Highland Terrier) and cattle dogs (25.0%, 5/20), followed by crossbreed dogs (2/20) and range of single dogs of other breeds (Border Collie, Dachshund, Labradors, Pointer, Shih Tzu). Forty percent (8/20) of dogs were between one and two years old, and 30.0% (6/20) were between three and five or between six and ten years old, respectively.

**Figure 2:**
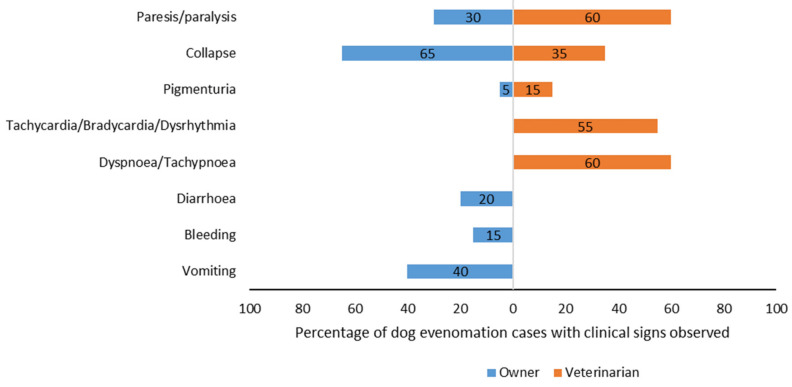
Recorded owner observations of dogs with snake envenomation vs. signs observed by examining veterinarians after presentation of these dogs to veterinary clinics in Queensland, Australia, in 2016.

Recorded owner observations indicated that signs of the envenomation were mostly noted after 8 a.m. and before 6 p.m. (65.0%, 13/20). Envenomation occurred in 80.0% (16/20) of dogs in the backyard of houses, followed by pastures and bushland (10.0%, 2/20), respectively. A snake was sighted in two cases. The most common signs of snake envenomation observed by owners were collapse and paresis/paralysis (Figure 2).

Paresis/paralysis, dyspnoea or tachypnoea, and cardiac abnormalities (such as dysrhythmias including tachycardia and bradycardia) were the most commonly reported clinical signs observed by veterinarians (Figure 2), with a combination of both respiratory and cardiac abnormalities observed in 10 dogs. Owners reported that an apparent recovery was observed for 40.0% of dogs (8/20) after the onset of initial clinical signs, with six dogs initially showing collapse, one showing paresis/paralysis and collapse, and one showing paresis/paralysis only. Seven of these achieved a full recovery, and one died. Veterinarians specified identification of the bite site in four dogs. The mean (SD) time between onset of clinical signs and admission to the veterinary hospital was 89.9 (95.1) minutes and the median (minimum, maximum) time was 60 (14, 360) minutes.

The diagnostic tests performed by veterinarians for suspected snake envenomation are shown in Table 1. Activated clotting time (ACT) was frequently combined with other diagnostic procedures. Prolonged clotting times were observed in 69.2% (9/13) of dogs where an ACT was performed. History and clinical signs were the sole diagnostic modalities utilised to determine snake envenomation in 30.0% of dogs (6/20).

An SVDK was used for 15.0% of dogs (3/20), with blood used as the test sample in two and urine in one dog. Results from the SVDK were the identification of one brown snake immunotype, one tiger snake immunotype, and one sample showing only a result for the positive control (thus, unable to confirm snake envenomation).

The veterinary treatments conducted for snake envenomation are shown in Table 2. Intravenous (IV) fluids were administered to 95.0% of dogs (19/20).

Antivenom was administered to 85.0% of dogs (17/20). Of the dogs that received antivenom, 13 dogs received a tiger/multibrown combination (3000 units tiger/4000 units brown), two dogs received a single multibrown (1650 units), one dog received two single multibrown, and one dog received taipan antivenom.

Of the three dogs where antivenom was not administered, one dog did not show a positive result for a snake immunotype using the SVDK and subsequently died. Another dog died before the treatment could commence, and the third dog showed pigmenturia and paralysis and then fully recovered.

Duration of hospitalisation (including for animals that died or were euthanised) was less than one day for 35.0% of dogs (7/20), one to two days for 30.0% (6/20), two to three days for 25.0% (5/20), three to four days for 5.0% (1/20), and greater than 4–6 days for 5% (1/20) of dogs. Twenty-five percent (5/20) of dogs were euthanised or died, while 75.0% (15/20) recovered following hospitalisation and treatment. Three of the 17 dogs that received antivenom (17.6%) died or were euthanised, while two of the dogs that not received antivenom (66.7%) died or were euthanised (Fisher exact *p* = 0.140).

## 4. Discussion

Based on extrapolation from 80 veterinary clinics surveyed in 1998, it was estimated that each veterinary clinic in Australia sees on average of four snake bite cases per year [6]. Our case series from 19 veterinary clinics indicated a higher snake envenomation rate of about 12 dogs per clinic, per year. Dogs with a hunting nature (e.g., terriers) were unsurprisingly more frequently bitten, but also younger dogs that are potentially more inquisitive or active. This is similar to the demographic trends reported in cane toad intoxication in dogs, where young terrier breeds are more frequently affected [13]. Animals with these characteristics are more likely to approach and attack snakes (and these breeds are very popular in Queensland, Australia), and possibly sustain multiple envenomations from agitated snakes. In contrast, dogs bitten by *Vipera berus* in Sweden were predominantly large breeds such as German shepherds and Labradors, which are actually the most popular breeds in this country [14].

In most cases, the owners of the dogs did not witness the envenomation and were therefore unable to describe the snake species. It has been reported previously, that even when a snake is witnessed, identification of snakes in Australia is challenging due to the morphologic variations, as well as due to the overlapping appearance between snake species [15]. Morrison et al. [16] highlighted that as few as 19% of snakes were correctly identified by the general population of Australians. Hence, relying on owners for visual identification of snakes to aid the diagnosis and treatment of snake envenomation is unreliable.

The seasonality of envenomation shown here is in accordance with previous research, which demonstrated peaks of envenomation in the warmer months of the year when snakes are active [17]. The seasonality of snake activity should increase the suspicion of envenomation when consistent clinical signs are present, but envenomation cannot be excluded outside these peak periods.

Previous snakebite investigations in dogs and cats highlighted that 78% of envenomations occurred in rural and 22% in urban areas [6]. The majority of snake envenomations in our case series occurred in backyards. Mirroring this, a recent report in people compiled from 171 hospitals in Australia demonstrated that the most frequent location for envenomation of 1548 people presenting for snake bite occurred near houses (485 people, 31%) and in buildings (220 people, 14%) [5]. The high frequency of envenomations in backyards in our case study might also be a reflection of companion animals spending their majority of time around their owner’s homes.

Clinical signs of envenomation were noted predominantly during the daytime, which is the main period of snake activity. Some elapids, especially eastern brown snakes (*Pseudonaja textilis*) and red-bellied black snakes (*Pseudechis porphyriacus*) are usually active during the day unless temperatures are very hot, and they are the most common venomous snakes to be encountered in Queensland [18,19].

Vomiting and collapse associated with a transient recovery as identified by owners in our study has been previously described as preparalytic signs [20,21], which is likely related to a sudden decrease in blood pressure [22]. The preparalytic phase is followed by the paralytic and lethal phase, which consists of skeletal muscle paralysis and coagulopathy [23].

Prolonged activated clotting time (ACT) was the most frequently performed test in our case series, with similar coagulopathy compared to previous research [15]. The presence of a coagulopathy in conjunction with preparalytic signs or paralytic signs should prompt the clinician to strongly consider snake envenomation as the diagnosis, but the absence of coagulopathy does not rule out envenomation. Pigmenturia was also observed in patients, which is due to either myolysis or haemolysis; thus, pigmenturia should prompt the veterinarian to consider snake envenomation [24]. While coagulopathy is a feature of many envenomations in small animals, spontaneous haemorrhage as a result is not. Three dogs were reported by owners to be bleeding prior to presentation, although details were not specified. Venom-induced consumptive coagulopathy may severely alter haemostatic capability, and any minor injury prior to presentation could potentially result in such clinically apparent haemorrhages.

Biochemical tests such as creatine kinase are beneficial in the determination of envenomation by snakes that cause myolysis such as tiger, black, and taipan snakes [7,25], but had only be used in two cases. The clinician should be aware that creatine kinase has a very short half-life of two hours and, depending on the cause of muscle damage, peaks around 4–12 h after insult [26].

The SVDK is manufactured by CSL Ltd. (Parkville, Victoria, Australia) and is able to detect the venom of the five major lethal snake immunotypes in Australia (brown, tiger, and black snakes; death adders; and taipans) [27]. The SVDK is designed for use in humans [28] but is also applied for the identification of venoms in companion animals [7,29]. The infrequent use of the SVDK in only three cases could be attributed to the cost (a single test costs about ~$300–400 AUD), as well as the varied accuracy of the results [15,24]. The SVDK was reported to give positive results in animals presented within four hours of envenomation but gave false negative results after delayed presentation [24]. The SVDK may yield negative results if venom in the urine has already been voided by the patient or if absorption of the venom to detectable levels has not occurred—we had one negative SVDK test result in our case series. The use of SVDK is common in humans (used in 83% envenomed patients [5]) with patients typically able to identify where they were bitten, as a bite-site swab is the preferred test sample for the SVDK. Conversely, bite sites in animals are unlikely to be identified, and therefore either urine or blood is used for the SVDK.

The majority of antivenom used was the tiger–multibrown antivenom, which contains 4000 units of multibrown and 3000 units of tiger snake antivenom. Reasons for this choice were not specified by veterinarians, although it may be preferred as the antivenom neutralises the venom of several species of snake such as brown, red-bellied black, and tiger snake, which are the most common snakes in the geographic area studied. In addition, the cost per unit is less for combination tiger–multibrown antivenom than for multibrown antivenom. Furthermore, due to the limited shelf life of antivenom, veterinarians may be more likely to stock combination antivenom, because of its versatility in application.

Unfortunately, we had a low response rate in our study, most likely as it is time consuming for veterinarians to provide detailed data on individual envenomation cases. The use of software applications that compile de-identified records from multiple veterinary clinics should be considered for future research on snake envenomation [30]. In addition, the use of mobile phone applications could provide an opportunity for researchers to obtain data directly from members of the public about snakes observed and potential envenomations of their pets.

Nevertheless, our case series has summarised the common diagnostic procedures and treatments of snake envenomation in dogs in Queensland. It seems that the dilemma faced by veterinarians compared to human medical practitioners is the daily balancing of costs associated with appropriate diagnostics and therapeutics. Thus, veterinarians are frequently reliant on history and clinical signs and diagnostic tests other than the SVDK (such as blood clotting times and serum CK) and most likely administered snake envenomation treatment based on their clinical experience.

## Figures and Tables

**Table 1:** Diagnostic procedures conducted by veterinarians for snake envenomation in dogs in Queensland, Australia, in 2016.

Diagnostic Procedures Performed	Dogs (%, n)
ACT only	35.0% (7)
ACT and PCV/TP	10.0% (2)
ACT and SVDK	10.0% (2)
ACT, PCV/TP, and blood smear	5.0% (1)
ACT, PCV/TP, and CK	5.0% (1)
SVDK, PTT/aPTT, and ECG	5.0% (1)
Clinical signs only	30.0% (6)
Total	100.0% (20)

ACT: activated clotting time, CK: creatine kinase, ECG: electrocardiograph, PCV/TP: packed cell volume/total protein, PTT/aPTT: prothrombin time/activated prothrombin time, SVDK: snake venom detection kit (SVDK).

**Table 2:** Treatments conducted by veterinarians for snake envenomation in dogs in Queensland, Australia, in 2016.

Treatment Administered	Dogs (%, n)
IV Fluids and Antivenom	35.0% (7)
IV Fluids, Antivenom, and Antihistamines	15.0% (3)
IV Fluids, Antivenom, Adrenalin, and Antihistamines	25.0% (5)
IV Fluids, Antivenom, Antihistamines, and Vitamin C	5.0% (1)
IV Fluids, Antivenom, Adrenalin, Frusemide, and Vitamin C	5.0% (1)
IV Fluids, Adrenalin, Frusemide, and Antihistamines	5.0% (1)
IV Fluids and Vitamin C	5.0% (1)
No treatment	5.0% (1)
Total	100% (20)

IV: intravenous.

## Data Availability

Data is contained within the article or supplementary material.

## Supplementary Materials

The following materials are available online at https://www.mdpi.com/2306-7381/8/2/14/s1, Figure S1. Online form used for collecting data on snake envenomations in dogs in Queensland, Australia, in 2016. Figure S2. Number of snake envenomations in dogs submitted to veterinary clinics in Queensland, Australia, in 2016. Data are shown by the postcode of veterinary clinics.
