# Supplementary material for: Diagnosis and Treatment of Snake Envenomation in Dogs in Queensland, Australia
Source: Vet Sci. 2021 Jan 20;8(2):14. doi: 10.3390/vetsci8020014 (PMC7909414; doi:10.3390/vetsci8020014)
Supplement: Supplementary file 1 [file vetsci-08-00014-s001.pdf]

Background information and clinic details

You are about to undertake a survey exploring the prevalence of, and the clinical and pathological characteristics for elapid snake envenomation of small animals in Queensland, Australia.

Your views are very important to us!

This project aims to fill the knowledge gap of the prevalence and geographical distribution of small animal elapid snake envenomation in the state of Queensland, Australia. Data will be collected on assumed or confirmed snake bite cases presented to animal veterinary clinics. This study is vital for a better understanding of the clinical signs and pathological findings associated with various venomous snake species, and will in turn allow for improved accuracy of diagnosis.

We would like to invite you to participate in this survey conducted by the University of Queensland. Please read the participant information sheet accompanying this form and complete the consent form below.

Your participation is voluntary. All information from the survey will remain confidential to the research team. None of the information that I provide will be described or portrayed in any way that will be identify me in any report on the study

This study adheres to the Guidelines of the ethical review process of The University of Queensland and the National Statement on Ethical Conduct in Human Research. Whilst you are free to discuss your participation in this study with project staff (contact details below), if you would like to speak to an officer of the University not involved in the study, you may contact the Ethics Coordinator on 3365 3924.

Principal Investigator
Ludovica Valenza, Ph: 0439828702
Email: ludovica.valenza@uqconnect.edu.au

PLEASE ENSURE THAT YOU READ AND ANSWER ALL THE QUESTIONS ON THIS SURVEY .

THANK YOU VERY MUCH FOR YOUR COOPERATION IN THIS IMPORTANT STUDY TO INVESTIGATE SNAKE ENVENOMATION IN OUR COMPANION ANIMALS.

PLEASE CLICK THE FOLLOWING BOX TO AGREE TO PARTICIPATE IN THIS SURVEY.

* 1. I agree to participate in this survey.

- ○ Yes

2. Name of veterinarian

[ ]

3. Name of clinic

[ ]

4. Postcode of clinic

5. Phone number (only used to follow-up on questions, if necessary)

Snake envenomation cases over the past 12 months

6. Please list the number of snake envenomation cases you have observed in your clinic over the past 12 months?

	Dogs	Cats
Jan 16		
Feb 16		
Mar 16		
Apr 16		
May 16		
Jun 16		
Jul 16		
Aug 16		
Sep 16		
Oct 16		
Nov 16		
Dec 16		

Information on the last snake two envenomation patients - patient 1

Patient 1 - Patient and envenomation details

7. Species of patient 1.

8. Age (years) of patient 1

9. Gender of patient 1.

10. Breed of patient 1.

11. Post code where envenomation took place for patient 1.

12. Date and time of envenomation (if known) for patient 1.

Date/ Time

DD/MM/YYYY hh mm -

13. Location where envenomation took place (if known) for patient 1.

- Inside the house
- Urban area
- Open woodlands
- Backyard
- Creek/near water
- Grass/pasture
- Other location (please specify)

14. Patient's signs observed by *owner* for patient 1.

- ☐ Vomiting
- ☐ Bleeding
- ☐ Blood in the urine
- ☐ Diarrhoea
- ☐ Paresis/paralysis
- ☐ Collapse
- ☐ Apparent recovery
- ☐ Other symptoms observed by owner (please specify)

15. Time after envenomation when signs were observed in patient 1 (hours).

- ○ <1
- ○ 1-2
- ○ 3-4
- ○ 5-7
- ○ 8-14
- ○ >14
- ○ unknown

Information on the last snake two envenomation patients - patient 1

Patient 1 - Veterinary examination

16. Date and time when patient 1 was admitted to the veterinary hospital.

Date / Time

DD/MM/YYYY hh mm -

17. Clinical signs observed by the veterinarian in patient 1.

- [ ] Collapse
- [ ] Dyspnoea/tachypnoea
- [ ] Tachycardia/Bradycardia/Dysrrhythmia
- [ ] Paralysis/paresis
- [ ] Pigmenturia
- [ ] Bite site identification
- [ ] Other clinical signs observed (please specify)

18. Diagnostic tests performed on patient 1.

- [ ] Blood smear
- [ ] Activated clotting time (ACT)
- [ ] PT/aPTT
- [ ] Whole Blood Tube Clotting time (>20kg)
- [ ] Snake Bite Detection Kit (see below)
- [ ] Other diagnostic tests performed (please specify)

19. Please indicate what body fluid you used from patient 1 for the Snake Venom Detection Kit (SVDK).

- ○ Blood
- ○ Urine
- ○ Both

20. Please provide the test results of the Snake Venom Detection Kid (SVDK) for the blood sample of patient 1.

- [ ] Well 1: Tiger Snake Immunotype
- [ ] Well 2: Brown Snake Immunotype
- [ ] Well 3: Black Snake Immunotype
- [ ] Well 4: Death Adder Immunotype
- [ ] Well 5: Taipan Immunotype
- [ ] Well 6: Positive Control
- [ ] Well 7: Blank Well

21. Please provide the test results of the Snake Venom Detection Kid (SVDK) for the urine sample of patient 1.

- [ ] Well 1: Tiger Snake Immunotype
- [ ] Well 2: Brown Snake Immunotype
- [ ] Well 3: Black Snake Immunotype
- [ ] Well 4: Death Adder Immunotype
- [ ] Well 5: Taipan Immunotype
- [ ] Well 6: Positive Control
- [ ] Well 7: Blank Well

22. Please summarize the other diagnostic test results (not SVDK) for patient 1.

23. Treatments performed on patient 1.

- [ ] Frusemide
- [ ] IV fluids
- [ ] Vitamine C
- [ ] NSAIDS
- [ ] Adrenalin
- [ ] Anti-histamines
- [ ] Antivenom (see below)

24. Details on Antivenom used in patient 1.

Type of antivenom used

Number of units used (if known)

Number of vials used

25. Duration of hospitalisation (days) in patient 1.

- ( ) 0-1
- ( ) 1-2
- ( ) 2-3
- ( ) 3-4
- ( ) 4-6
- ( ) >7

26. Outcome for patient 1.

- ◯ Passed away/euthanised
- ◯ Full recovery
- ◯ Partial recovery
- ◯ Unknown

27. Date when passed away/euthanised for patient 1.

Date / Time

DD/MM/YYYY

Information on further snake two envenomation patients - patient 2 - 5

Supplementary Figure S1. Online form used for collecting data on snake envenomations in dogs in Queensland, Australia, in 2016.

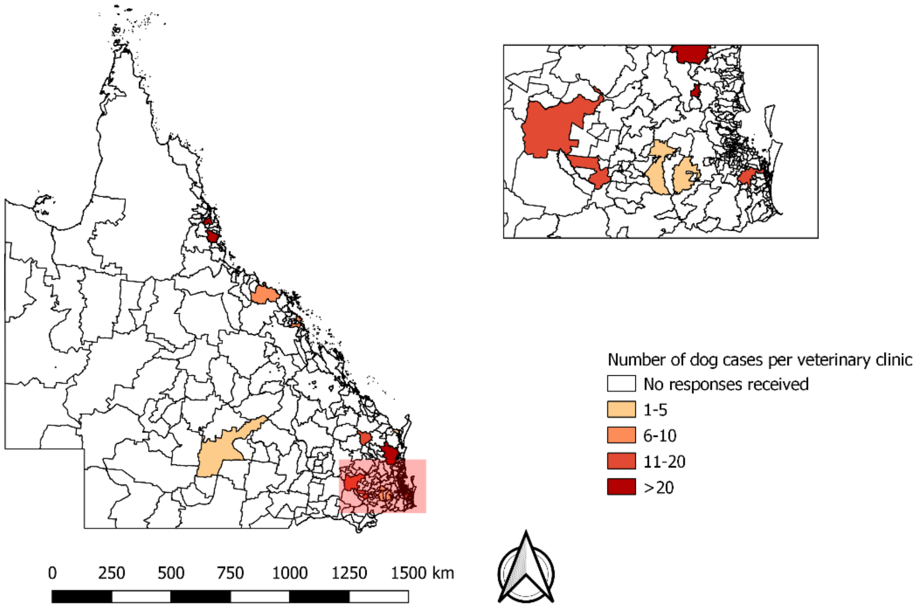

Supplementary Figure S2. Number of snake envenomations in dogs submitted to veterinary clinics in Queensland, Australia, in 2016. Data is shown by postcode of veterinary clinics.
